# Historical Sketch of the Use of the Affusion of Cold Water

**Published:** 1800-11

**Authors:** Henry Reeve

**Affiliations:** Student


### *Historical Sketch of the Use of the Affusion of Cold Water.*

*By* Henry Reeve, Student.

" In quibus explicandis non dubitabo auctoritate antiquorum virorum uti, maximèque Hippocratis, cùm recentiores quoque medici, quamvis quædam in curationibus mutarint, tamen hæc illum optimè præsagisse fateantur."

Celsus.

FROM the writings of learned men, and the most authentic historical records, it appears, that no nation, however barbarous and uncivilized, has yet been discovered totally unacquainted with diseases and the knowledge of remedies. The various circumstances that might lead to the discovery of medicinal virtues and powers, are with difficulty ascertained; but the learned Dr. Cullen has suggested, that the rude and ignorant must have been directed to the invention of remedies by the instincts arising in certain diseases, by accidental observation, or by random trials, to which pain and uneasiness often lead. It seems probable, therefore, that cold water has been in the list of remedies among all nations from the earliest ages. The use of water as a common drink, and its being so well adapted by Nature for the various purposes of the animal œconomy, must soon have attracted attention for the cure of diseases; and as bathing has been universally practised, experience has proved that the external use of it is no less beneficial and safe.

If we look into the old writers on physic, we shall find that cold water has been recommended and employed in fevers in the most ancient times: And it might have been expected, from its well-known properties and good effects, that the use of it would have continued, and have been brought into general practice; but it has sometimes been highly extolled, and at another time almost totally neglected, which must be imputed to practitioners in physic searching after more compound remedies, and therefore despising the simplicity of water; and moreover, to their observing it prove very useful in some instances, yet attended with pernicious consequences from misapplication in other cases.

These trifling observations were occasioned by reading the following remark in a late classical and ingenious publication "On the Effects of Water in Fevers and other Disorders," by Dr. Currie. After enumerating the effects and advantages of the affusion of cold and warm water, in chap. x. p. 75, the Doctor goes on to say; "The practice of giving cold water as a drink in fevers, was common among the ancients; and immersion immerſion in cold water they occaſionally employed, but the *affuſion of it* on the ſurface of the body ſeems to have been *wholly unknown.* Ablution of the ſurface with cold water was firſt practiſed in modern times at Breſlaw, in Sileſia, as appears from a diſſertation, by I. G. de Hahn, under the title of Epidemia verna quæ Wrateſlaviam, Anno 1737, afflixit."

It would be needleſs to cite the numerous inſtances that might be adduced of the uſe of cold water in fevers, inflammations, &c. from the oldeſt authors; and as the external uſe of it, in the way of ablution and affuſion, ſeems to have been doubted, and not generally known, I ſhall humbly attempt to prove that it was both known and practiſed long before the epidemic in Sileſia.

The firſt author who notices the uſe of water in diſeaſes, together with almoſt every thing important to the ſcience of medicine, is Hippocrates, who appears to have been a ſtrenuous advocate for the uſe of it, both as an internal and external remedy. It may be remarked, however, that Hippocrates in his account of epidemics, which is wholly employed in treating upon fevers, delivers the particular hiſtory of the diſeaſe, and rarely mentions the remedies. We are therefore not able confidently to decide, whether he always uſed the cold affuſion in caſes of fever; although we may conclude that it was not neglected or diſregarded by him, ſince we find in Caſe 7, book 1, the patient drank largely of cold water, and had it poured upon his head, which moderated the delirium, and he became rational and recovered, having at the ſame time a critical hæmorrhage from the noſe. Sir John Floyer, in his Pſychroluſia, or Hiſtory of Cold Bathing, has obſerved, that Hippocrates deſcribes, in his Aphoriſms, the virtues of hot and cold water, without mentioning affuſions, fomentations, or baths; but the το ψυχρὸν or το θερμὸν, relate to all of them equally. The term uſed by Hippocrates is καταὰκλυσμος or καταχυσις, which ſignifies perfuſion, or affuſion, and was performed by a ſervant, who poured the water upon thoſe perſons who were recommended to try its effects in various diſeaſes; and the ſame virtues are aſcribed by him to this method as to cold baths. If the internal uſe of cold water was only known to Hippocrates, he would not have given directions about affuſions, lotions, and fomentations, as he has done in his tracts upon the uſe of liquids, and upon the diet in acute diſeaſes; and eſpecially as the latter part of the tract De liquidorum uſu, is entirely upon the effects of καταχυσις, or affuſion. Beſides, it ſeems probable that he was well acquainted with the neceſſary cautions to be attended to in applying the affuſion, ſince, to ſupply the deficiency of thermometrical obſervations, he adviſes the ſkin of the patient, or or of the perſon who pours on the water, to be the criterion of the degree of cold or heat; and he cautions againſt proceeding to any great exceſs, which might prove injurious. In the cure of typhus he adviſes to refrain from immerſion for the firſt few days, but recommends cloths wetted with cold water to be applied where the patient complains moſt of heat; which method anſwers to the "lavatio frigida," as practiſed by Dr. Gregory at Edinburgh. Hippocrates, after mentioning the advantages of drinking and bathing in cold water, obſerves that it produces more powerful effects by affuſion, δυνατωτερον καταχειν; and as he has ſtudiouſly avoided the appearance of empiriciſm by combining reaſoning with events, he thought the cold water produced heat and ſweat, and that the heat cured the diſeaſes for which the uſe of water was moſt effectual.

Although Aſclepiades, Celſus, Galen, and many other old authors, have noticed the uſe of cold water, it does not appear that they generally underſtood the affuſion of it upon the ſurface of the body, or that ſuch a mode of applying it was in great repute among them. Yet we find Aretæus, in his chapter De curatione Phreniticorum, adviſes the liberal affuſion of cold water upon the patient; and Galen alſo practiſed ablution in ardent fevers; and in Lib. x. de Methodo Medendi, he has laid down rules for the proper application of it. And other writers have recommended in vertigo and inveterate head-achs, "ut caput frigidâ aquâ perfundant." The antiquity of the external application of cold water may perhaps be further illuſtrated by the relation of Auguſtus Cæſar's caſe, as mentioned in his life by Suetonius: "Cum etiam diſtillationibus jecinore vitiato ad deſperationem redactus, contrariam et ancipitem rationem medendi neceſſario ſubiit, quia calida fomenta non proderant, frigidis curari coactus, auctore Antoniâ Muſâ."—Sueton. lib. ii.

Hiſtory informs us that the American Indians have always practiſed cold immerſion for the cure of fevers, to which they are particularly ſubject; nor is this practice confined to warm climates, ſince the northern nations make uſe of that cuſtom both for the prevention and cure of diſeaſes. The affuſion and ablution of the body might firſt take its origin from the cuſtom of purifying the body with water, in great eſteem among the patriarchs, and imitated from them by the Egyptians, Greeks, and Romans; and the uſe of it, probably, became more general at the introduction of Chriſtianity, when the ceremony of baptiſm was univerſally practiſed by what was called the trine immerſion, or by placing the perſons in the font and pouring water on their heads and bodies three times. In a work publiſhed about the beginning of the preſent century, entitled

 Pſychroluſia,

Pfychrolufia, or Hiftory of Cold Bathing, by Sir John Floyer and Dr. Baynard, the ufe of cold water applied to the furface of the body is much recommended and infifted upon for the cure of almoft all difeafes; and although that book partakes too much of what would juftly be called medical enthufiafm, yet it contains many important facts and ufeful obfervations. It feems rather remarkable that Dr. Currie fhould not have referred to this book among others which he has noticed, fince it would have furnifhed fome ftriking facts of no fmall confequence to his ingenious theory and judicious practice. Dr. Baynard mentions many cafes of perfons who have leaped into a pond, or any other water, in their delirium from fevers, and not one ever received any harm, but were thereby prefently cured. And he adduces inftances of maniacal perfons being plunged into cold water, and having ten or twelve pails of water thrown over them during the paroxyfm of infanity; and refers to a remarkable cafe related by Dr. Willis, in his Chapter de Delirio & Phrenitide, where the fame means were ufed with equally good fuccefs. No other work of importance, concerning the application of cold water to the human body, appeared till the year 1785, when an ingenious effay was publifhed by Mr. Rigby, of Norwich, " On the Theory and Production of Animal Heat, and its Application in the Treatment of Difeafes. As far as relates to the fimple abftraction of heat from the furface, the Author of that Effay feems to have faid as much as has been fince repeated by Dr. Currie and others; and the obfervations it contains upon the treatment of cutaneous difeafes, (efpecially fmall-pox, fcarlatina, and meafles, and local inflammations) are valuable, and defervedly claim attention. Hence it appears, that the external ufe of cold water has been known and practifed from the earlieft periods down to the prefent time; and this practice has not arifen as the mere fuggeftion of hypothefis, or the product of fpeculative inquiry, but has been eftablifhed and confirmed by long experience. Yet, after all that can be found in ancient authors upon the affufion in fevers and other difeafes, it will be readily acknowledged that their practice was unconfirmed, and the conclufions drawn from their experience were vague and uncertain. And it will be as readily acknowledged, that we are greatly indebted to Dr. Currie, who, by a diligent inveftigation, conducted with judgment and accuracy, has corrected the errors and fupplied the defects of preceding writers, and has been a valuable agent in eftablifhing the ufe of a remedy in the art of medicine, endued with the moft efficacious properties, and admirably calculated to produce the greateft benefit to all mankind.

*Edinburgh, Oct.* 15, 1800.

